# Brensocatib in non-cystic fibrosis bronchiectasis: targeting neutrophil-driven inflammation through DPP-1 inhibition

**DOI:** 10.1097/MS9.0000000000005111

**Published:** 2026-05-15

**Authors:** Sakan Binte Imran, Ehtisham Haider, Bisma Faraz, Pinkey Kumari

**Affiliations:** a Internal Medicine Department, Sir Salimullah Medical College Mitford Hospital, Dhaka, Bangladesh; b Medicine Department, Punjab Medical College, Punjab, Pakistan; c Karachi Medical and Dental College (KMDC), Karachi, Pakistan; d Medicine Department, Hamdard College of Medicine and Dentistry, Karachi, Pakistan

Non-cystic fibrosis bronchiectasis (NCFB) is a chronic respiratory disease characterized by irreversible bronchial dilation, chronic cough, sputum production, and recurrent exacerbations, leading to a progressive decline in lung function and quality of life. The disease burden is increasing globally. In the United States, the prevalence of NCFB is estimated at approximately 500 000 individuals, with rates rising markedly with age, from about 7 per 100 000 in adults aged 18–34 years to over 800 per 100 000 in those aged 75 years or older^[^1^]^. This letter to the editor is in compliance with the TITAN guideline 2025^[^2^]^.

Current management strategies include long-term antibiotics, inhaled therapies, airway clearance, and macrolide maintenance therapy. However, inhaled corticosteroids have not demonstrated consistent benefits in reducing exacerbations or improving lung function in bronchiectasis and are not routinely recommended unless there is coexisting asthma or other indications^[^3^]^. Long-term macrolide therapy has been shown to reduce exacerbation frequency, but its use is associated with adverse effects such as gastrointestinal intolerance, ototoxicity, and the risk of antimicrobial resistance, and the optimal duration remains uncertain^[^1^]^. Given these limitations, there is a growing interest in therapies targeting the underlying neutrophilic inflammation that drives airway damage and disease progression. Brensocatib is an oral, first-in-class, selective, reversible inhibitor of dipeptidyl peptidase-1 (DPP-1), an enzyme responsible for activating neutrophil serine proteases such as neutrophil elastase, proteinase 3, and cathepsin G^[^4^]^. By inhibiting DPP-1, brensocatib reduces neutrophil protease activity and downstream tissue damage, thereby targeting a central mechanism in bronchiectasis pathophysiology^[^4,5^]^.

Exacerbations are a hallmark of NCFB and are characterized by worsening respiratory symptoms requiring treatment modification. Patients colonized with *Pseudomonas aeruginosa* are at particularly high risk of frequent exacerbations, worse clinical outcomes, and increased healthcare utilization^[^1^]^.

Brensocatib has recently been approved by the U.S. Food and Drug Administration as the first therapy specifically indicated for NCFB, representing a significant advancement beyond traditional symptomatic management approaches^[^6^]^. In the phase 2 WILLOW trial, both 10 and 25 mg doses of brensocatib significantly prolonged the time to the first pulmonary exacerbation and reduced annualized exacerbation rates compared with placebo (approximately 36 and 25% reduction, respectively)^[^7^]^. The treatment was generally well-tolerated, with most adverse events being mild to moderate^[^5,7^]^. More recently, the phase 3 ASPEN trial further confirmed reductions in exacerbation rates and demonstrated clinically meaningful benefits, including effects on lung function and sustained disease control over a longer follow-up period^[^8^]^.

Despite these promising findings, several important questions remain. The long-term safety and immunological consequences of sustained DPP-1 inhibition require further evaluation, particularly regarding potential impacts on host defense and infection risk^[^4,8^]^. Additionally, optimal patient selection and dosing strategies are yet to be fully established, and future studies should focus on identifying clinical or biomarker-based predictors of response to enable more personalized therapy^[^7,8^]^.

Beyond bronchiectasis, DPP-1 inhibition may have broader therapeutic implications in other neutrophil-driven airway diseases, including chronic obstructive pulmonary disease and severe asthma, although further research is required to establish efficacy in these conditions^[^4^]^.

In conclusion, brensocatib represents a significant step forward in the management of NCFB. By targeting neutrophil-driven inflammation, it offers a mechanism-based therapeutic approach with demonstrated clinical benefits. Continued research is needed to confirm long-term safety, refine patient selection, and optimize its role in clinical practice.

## Data Availability

Upon request.

## Permission to reproduce material from other sources

Not applicable. No third-party material was reproduced.
